# Effects of Incorporating Carboxymethyl Chitosan into PMMA Bone Cement Containing Methotrexate

**DOI:** 10.1371/journal.pone.0144407

**Published:** 2015-12-14

**Authors:** Bo-Ming Liu, Ming Li, Bao-Sheng Yin, Ji-Yang Zou, Wei-Guo Zhang, Shou-Yu Wang

**Affiliations:** 1 Department of Orthopedics, First Affiliated Hospital of Dalian Medical University, Dalian, Liaoning, 116044, China; 2 Dalian Medical University, Dalian, Liaoning, 116044, China; Wayne State University School of Medicine, UNITED STATES

## Abstract

Treatment of bone metastases usually includes surgical resection with local filling of methotrexate (MTX) in polymethyl methacrylate (PMMA) cement. We investigated whether incorporating carboxymethyl chitosan (CMCS) in MTX-PMMA cement might overcome disadvantages associated with MTX. To determine the optimal CMCS+MTX concentration to suppress the viability of cancer cells, an integrated microfluidic chip culturing highly metastatic lung cancer cells (H460) was employed. The mechanical properties, microstructure, and MTX release of (CMCS+MTX)-PMMA cement were evaluated respectively by universal mechanical testing machine, scanning electron microscopy (SEM), and incubation in simulated body fluid with subsequent HPLC-MS. Implants of MTX-PMMA and (CMCS+MTX)-PMMA cement were evaluated *in vivo* in guinea pig femurs over time using spiral computed tomography with three-dimensional image reconstruction, and SEM at 6 months. Viability of H460 cells was significantly lowest after treatment with 57 μg/mL CMCS + 21 μg/mL MTX, which was thus used in subsequent experiments. Incorporation of 1.6% (w/w) CMCS to MTX-PMMA significantly increased the bending modulus, bending strength, and compressive strength by 5, 2.8, and 5.2%, respectively, confirmed by improved microstructural homogeneity. Incorporation of CMCS delayed the time-to-plateau of MTX release by 2 days, but increased the fraction released at the plateau from 3.24% (MTX-PMMA) to 5.34%. Relative to the controls, the (CMCS+MTX)-PMMA implants integrated better with the host bone. SEM revealed pores in the cement of the (CMCS+MTX)-PMMA implants that were not obvious in the controls. In conclusion, incorporation of CMCS in MTX-PMMA appears a feasible and effective modification for improving the anti-tumor properties of MTX-PMMA cement.

## Introduction

The most common site of cancer metastasis is the bone. Metastatic cancer of the bone is traditionally treated by surgical resection, and methotrexate (MTX) mixed within polymethyl methacrylate (PMMA) bone cement is applied as the local filler [1–3]. Although PMMA bone cement with MTX provides satisfactory outcomes, the method also has disadvantages that limit clinical success. The high local concentrations of MTX are associated with toxic effects and delayed wound healing [4]. The MTX is usually released quickly and its anti-tumor activity cannot be sustained [5]. Furthermore, because only a small fraction of the MTX in the cement is released, clinicians frequently increase its concentration, aggravating the adverse side effects and compromising the mechanical properties of the cement [5]. The conventional PMMA bone cement has a dense structure that limits bone ingrowth, and due to poor cement-bone integration, the cement can gradually loosen [6–7].

Carboxymethyl chitosan (CMCS) is a natural polysaccharide with excellent water solubility, ability to form films [8–11], and is biosafe [12]. Moreover, CMCS is a derivative of chitosan, a well-known osteoconductive material [13, 14], and therefore is also likely to be osteoconductive. Recent studies found that CMCS is associated with anti-tumor activity [15] and can increase the host immune response [16–18]. Consequently, this polysaccharide is finding an increasing number of biomedical applications.

There is limited understanding of the effects of CMCS on MTX and PMMA cement, and no study has explored how CMCS may modify the mechanical properties of the cement. We hypothesized that incorporating CMCS into MTX-containing PMMA cement (MTX-PMMA) may result in the co-release of CMCS and MTX. Perhaps the two compounds would have a synergistic effect in killing or inhibiting tumor cells, increase the proportion of MTX released from the cement matrix, modify the rate of MTX release, or reduce the adverse side effects of MTX. Earlier studies reported that derivatives of chitosan can modify the mechanical performance of PMMA [19, 20]. Because CMCS is a polyelectrolyte with high viscosity [21], incorporating CMCS into PMMA cement may modulate the mechanical properties of the cured cement, making it suitable for various skeletal environments. Our objective in the current study was to investigate these possibilities.

A microfluidic chip (also known as a lab-on-a-chip) is a type of micro-electro-mechanical device which allows scaling one or more lab processes down to a chip format, including the analysis of micro volumes of fluids [22]. This technique offers several avenues for biomedical studies. For example, a concentration gradient can be formed on a chip, thereby allowing efficient screening of drug concentrations and combinations on cells. In the current study, we first used microfluidic chips to investigate the effectiveness of various concentrations of MTX and CMCS for killing H460 lung cancer cells. Noting the optimal concentrations of the combined drugs for killing H460 lung cancer cells, we then incorporated CMCS into MTX-PMMA cement at various concentrations to evaluate the mechanical properties of the cement and the amount and rate of MTX release. Finally, in guinea pig femurs cement implants containing MTX and CMCS were evaluated relative to implants containing MTX alone, with regard to microstructural changes and interfacial integration with the adjacent bone.

## Materials and Methods

### Determination of testing concentrations

The first stage of the study consisted of experiments to determine the optimal concentrations of MTX and CMCS for subsequent testing in PMMA cement. Microfluidic chips capable of creating mixtures of predetermined concentrations of microvolumes of MTX and CMCS were utilized, and lung carcinoma cells were then treated with these mixtures and tested for viability.

### Microfluidic chip and concentration gradient generator

**Fig 1:**
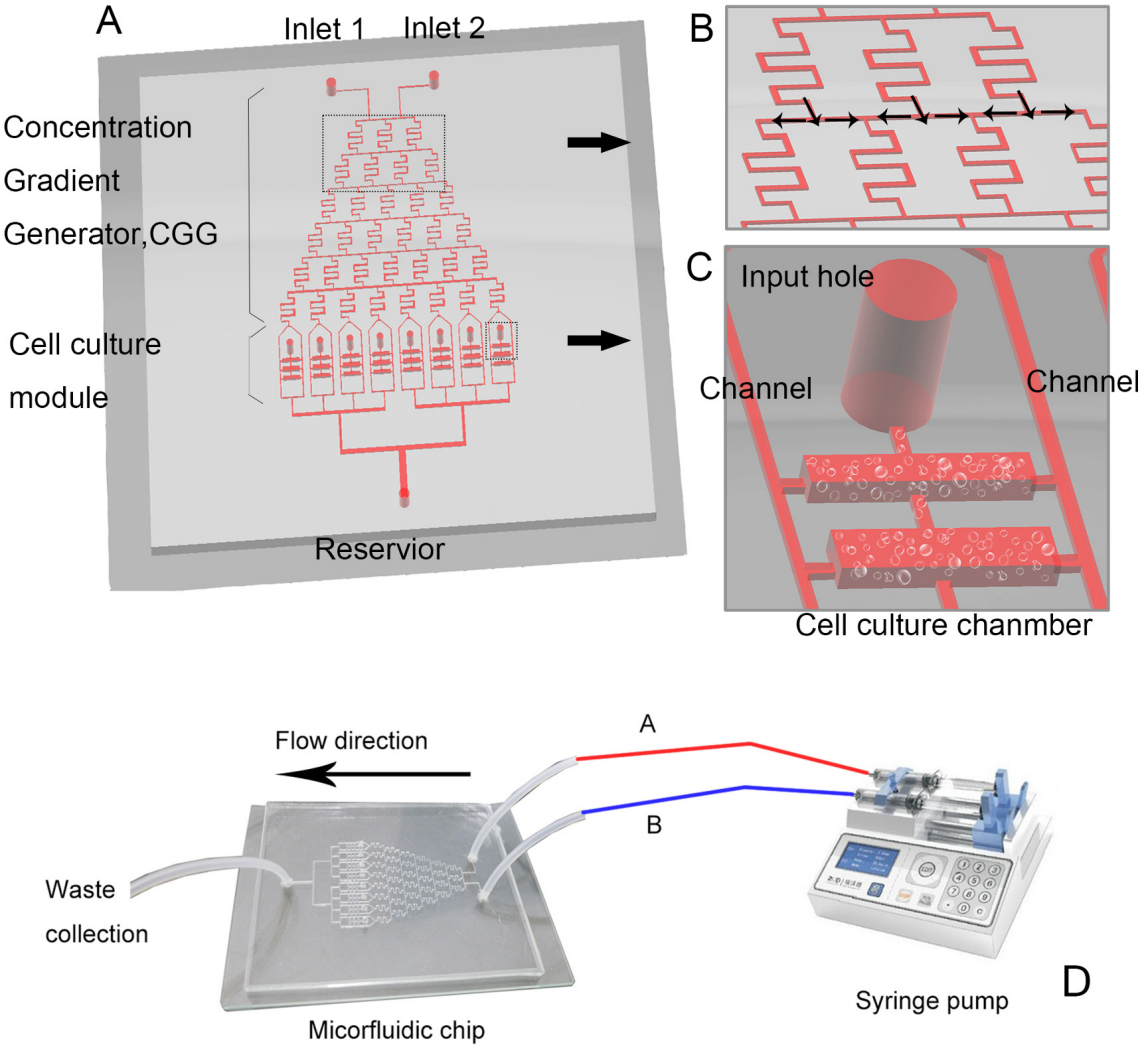
Design of (A) the microfluidic chip, (B) concentration gradient generator, and (C) cell culture chambers; (D) instrumentation of the microfluid device by connection of the chip to a syringe pump.

The miocrofluidic system was fabricated on a polydimethylsiloxane substrate via soft lithography, and attached to a glass plate (Fig 1). The miocrofluidic chip consisted of a concentration gradient generator (a network of channels) and a cell culture unit. The protocol required that, via two respective inlets, a syringe pump drives fluid A and fluid B, each carrying a target substance (*a* and *b*, respectively; see below), into a channel at 0.1 μL/min, to form laminar flows. When passing the concentration gradient generator, the two flows mix, separate, and dilute repeatedly to generate a stable concentration gradient at the outlets. According to a previously developed formula [23], our system was designed to deliver respectively the following mixed concentrations of the target substances at the eight outlets: *a*, 6/7*a* + 1/7*b*, 5/7*a* + 2/7*b*, 4/7*a* + 3/7*b*, 3/7*a* + 4/7*b*, 2/7*a* + 5/7*b*, 1/7*a* + 6/7*b*, and *b*.

In the present study, fluids A and B contained CMCS (*a*, 100 μg/mL) and MTX (*b*, 50 μg/mL), respectively (Table 1). To confirm the predicted concentrations, we used acridine orange (a 438.12-dalton fluorescent dye, excitation: 488 nm; Gibco, Carlsbad, CA, USA) as a surrogate as follows. An acridine orange solution and phosphate buffered saline were respectively pumped at 0.1 μL/min into the system via the two inlets. The concentrations of acridine orange at the eight outlets were fluorescently monitored and compared with predicted values according to Pearson’s correlation (SPSS12.0; SPSS, Chicago, IL, USA). The measured fluorescence intensities at the outlets agreed satisfactorily with the predicted values (see Results).

**Table 1:** Concentrations of CMCS (*a*) and MTX (*b*) at the eight outlet channels of the microfluidic chip, μg/mL.

Channel	Concentrations	CMCS	MTX
1	*A*	100	0
2	6/7*a* + 1/7*b*	86	7
3	5/7*a* + 2/7*b*	71	14
4	4/7*a* + 3/7*b*	57	21
5	3/7*a* + 4/7*b*	43	29
6	2/7*a* + 5/7*b*	29	36
7	1/7*a* + 6/7*b*	14	43
8	*B*	0	50

### On-chip cell culture and drug exposure

Highly metastatic human large-cell lung carcinoma cells (H460; KeyGen Biotech, Nanjing, Jiangsu, China) were seeded in 0.24% collagen gel (Gibco) and plated in a cell culture unit (0.5 μL of gel/chamber). After incubation (37°C, 1 h, 5% CO_2_) to stabilize the gel and allow cells to adapt to the environment, Dulbecco’s modified Eagle’s medium (Sigma, St Louis, MO, USA) containing 10% fetal bovine serum (Gibco) and penicillin/streptomycin (100 U/mL; Sigma) was gently pumped into the system. After incubation for 24 h, solutions of CMCS (100 μg/mL; Meilun Biotech, Dalian, Liaoning, China) and MTX (50 μg/mL; Lingnan Pharmaceutical, Guangzhou, Guangdong, China) were delivered (via two portals) into the system at constant rates, thereby generating the pre-designed concentration gradient.

After maintaining the gradient for 2 d, the viability of the H460 cells was examined by staining with acridine orange (6 μg/mL) and propidium iodide (10 μg/mL; both Gibco). The procedure is based on the following principle. Acridine orange enters both live and dead cells, staining all nuclei to produce a green fluorescence. Propidium iodide enters only dead cells (via defective membranes), staining dead cells with a red fluorescence. Thus, after co-staining with acridine orange and propidium iodide, live cells fluoresce green and dead cells fluoresce red. In the present study, acridine orange and propidium iodide solutions were slowly pumped into the system. After incubation for 5 min, cells were studied under a fluorescence microscope.

### Flask culture to study effects of drug exposure on H460 cell growth

After completion of the above on-chip culture experiments, further cellular studies were made by traditional flask culture. Four groups were included, based on the treatment supplemented in the culture media of the cells: CMCS (57 μg/mL CMCS), MTX (21 μg/mL MTX), CMCS+MTX (57 μg/mL CMCS + 21 μg/mL MTX), and the control (unsupplemented). In each group, cells were seeded in a 96-well plate (10^4^ cells/well). After incubation for 24 h, the assigned treatment drug was added to the medium. After further culture for 0–8 d, cell viability was measured by MTT assay (570 nm, BioRAd 680 microplate reader, Hercules, CA, USA) using commercial reagent (Sigma, St. Louis, MO, USA), and the growth curve was determined. The drug concentrations used in the experiments below were based on results from these on-chip cell culture experiments.

### Mechanical properties and microstructures of drug-loaded bone cement

The second stage of the study evaluated the mechanical properties of MTX-PMMA cement containing CMCS and the amount and rate of MTX release.

#### Mechanical properties

Six sets of bone cements consisting of various percentages of combined CMCS and MTX (experimental groups) or corresponding percentages of MTX alone (control groups), were prepared (Table 2), with 4 replicates in each group. Thus, 48 samples were prepared and studied. The drug concentrations of these groups were selected based on the results of an earlier study [22] and our previous experiments (above).

**Table 2:** Sample groups for mechanical tests and microstructural observations.

Group	Experimental group	Control group
1	0% CMCS + 0% MTX	0% MTX
2	0.8% CMCS + 0.3% MTX	0.3% MTX
3	1.6% CMCS + 0.6% MTX	0.6% MTX
4	2.4% CMCS + 0.9% MTX	0.9% MTX
5	3.2% CMCS + 1.2% MTX	1.2% MTX
6	4% CMCS + 1.5% MTX	1.5% MTX

Briefly, PMMA bone cement (Zimmer, Warsaw, IN, USA) powders were blended with an appropriate amount of the drug(s) (Table 2), mixed with the liquid component, and cast into a cylinder (diameter: 4.7 mm, length: 64 mm). The mechanical properties of the cylinders were determined by four-point bending on a universal mechanical testing machine (MTS810, MTS, Minneapolis, MN, USA). The bending modulus was calculated as:

$$E=\frac{\left(F_{1}-F_{2}\right)}{\left(\omega_{1}-\omega_{2}\right)}\frac{x}{12I}(-4x^{2}+3xl),$$

where *E* is the elastic modulus, *F* the load, ω is the deflection at the loading point, *x* the distance between the loading point and supporting points, *I* the second moment of area (i.e., for a circular cross section with a diameter of *d*, *I* = π*d*·4/64), and *l* is the length of the cylinder. *F*_1_ and *F*_2_ are corresponding loads at moments *t*_1_ and *t*_2_, respectively. Similarly, ω_1_ and ω_2_ are displacements of the loading point at *t*_1_ and *t*_2_, respectively.

Based on theories of material mechanics, the bending strength and compressive strength of the cylinders were calculated as:

bending strength:σ=M/W,

where *M* is the bending moment of the middle section of the cylinder, and *W* is the bending modulus *W* = π*d* ^3^ / 32

and

compressive strength:σ=F/S,

where *S* is the cross sectional area of the sample.

#### Microstructure

The mechanical properties of the cement mixtures are related to their microstructure. Therefore, for each group of cement mixtures (Table 2), a thin sample (5 × 5 mm) was prepared, sputter-coated with gold, and studied using scanning electron microscopy (SEM, JSM-6360LV, JEOL, Tokyo, Japan).

### Effect of CMCS incorporation on MTX release from bone cement

In further experiments, to evaluate the release *in vitro* of CMCS and MTX from PMMA bone cement, 8 cylindrical PMMA bone cement samples (diameter: 10 mm, length: 15 mm) were prepared, comprising 4 controls (containing 1.5% MTX alone) and 4 samples with 4% CMCS and 1.5% MTX (all w/w). Each sample was immersed in 50 mL of simulated body fluid [21] (37°C) for up to 12 d, and 20 μL of fluid was collected at predetermined intervals. The MTX concentrations in the collected fluid were analyzed via high-performance liquid chromatography coupled to mass spectroscopy (HPLC-MS). The HPLC conditions were: mobile phase 0.1% formic acid-acetonitrile (85:15; v:v), flow rate 0.4 mL/min, and a Hypersil ODS-C18 column (150 × 2.1 mm, internal diameter: 5 μm, room temperature; Elite HPLC, Dalian, Liaoning, China). The MS conditions were: an electrospray ionization source, positive ion mode, injection voltage 4600 V, temperature 500°C; ionization source gas-1, 0.20 MPa nitrogen; ionization source gas-2, 0.30 MPa nitrogen; collision gas pressure 0.20 MPa; declustering potential 35 eV; multiple reaction monitoring; transition m/z 455.2→ 308.2. MS results were converted to concentrations against an experimentally established calibration curve, and expressed as the proportion of cumulative release.

### *In vivo* evaluation of MTX-PMMA cement implants containing CMCS

For the third stage of this study, the microstructural changes and interfacial integration of MTX-PMMA cement implants containing CMCS were evaluated *in vivo*, relative to implants without CMCS.

The Animal Ethics Committee of Dalian Medical University (Permission No.: L2014006), approved the study, and all procedures were in accordance with the Guidance for Care and Use of Laboratory Animals (Ministry of Science and Technology of China, 2006).

### *In vivo* implantation

Four adult guinea pigs (Animal Experiment Center, Dalian Medical University, Dalian, Liaoning, China) were anesthetized by inhalation of ether. A small hole (diameter: 2 mm, depth: 3 mm) was drilled on the lateral epicondyle of each femur. The hole in the left femur was filled with control cement (containing 1.5% MTX only). The hole in the right femur was filled with cement containing 4% CMCS and 1.5% MTX. The wound was closed layer in layers. After operation, the wound was cleansed daily with iodophore and treated with erythromycin ointment. The animal was also given intraperitoneal injection of penicillin for 3 d (one injection of 80000 U /day). Each animal was maintained in a separate cage with free access to food and drinking water. The cage was cleaned daily and drinking water refreshed everyday. Each animal was monitored for food intake and defecation to assess its postoperative recovery. Two weeks after operation, the skin at the wound site has healed and the suture was removed.

### Evaluation of implants

After the operation, the animals were examined by spiral computed tomography (CT) and three-dimensional image reconstruction to evaluate the position of the cement filler. The operated limbs were examined on the day of the surgery, and at 1, 1.5, 3, and 6 months using radiography to evaluate the bone-cement interface.

Six months after the operation, the animals were uthanized by intraperitoneal injection of chloral hydrate (100 mg/kg). The bone-cement interface was examined by SEM under high-resolution imaging mode.

### Statistical analyses

The viability of H460 cells (cultured on chip and plate) and the mechanical properties of PMMA cement samples were analyzed by analysis of variance and post hoc tests (SPSS12.0; SPSS, Chicago, IL, USA). MTX concentrations measured during *in vitro* release were analyzed with the rank sum test. A *P*-value < 0.05 was considered statistically significant.

## Results

### Determination of testing concentrations

#### Concentration gradient verification

**Fig 2:**
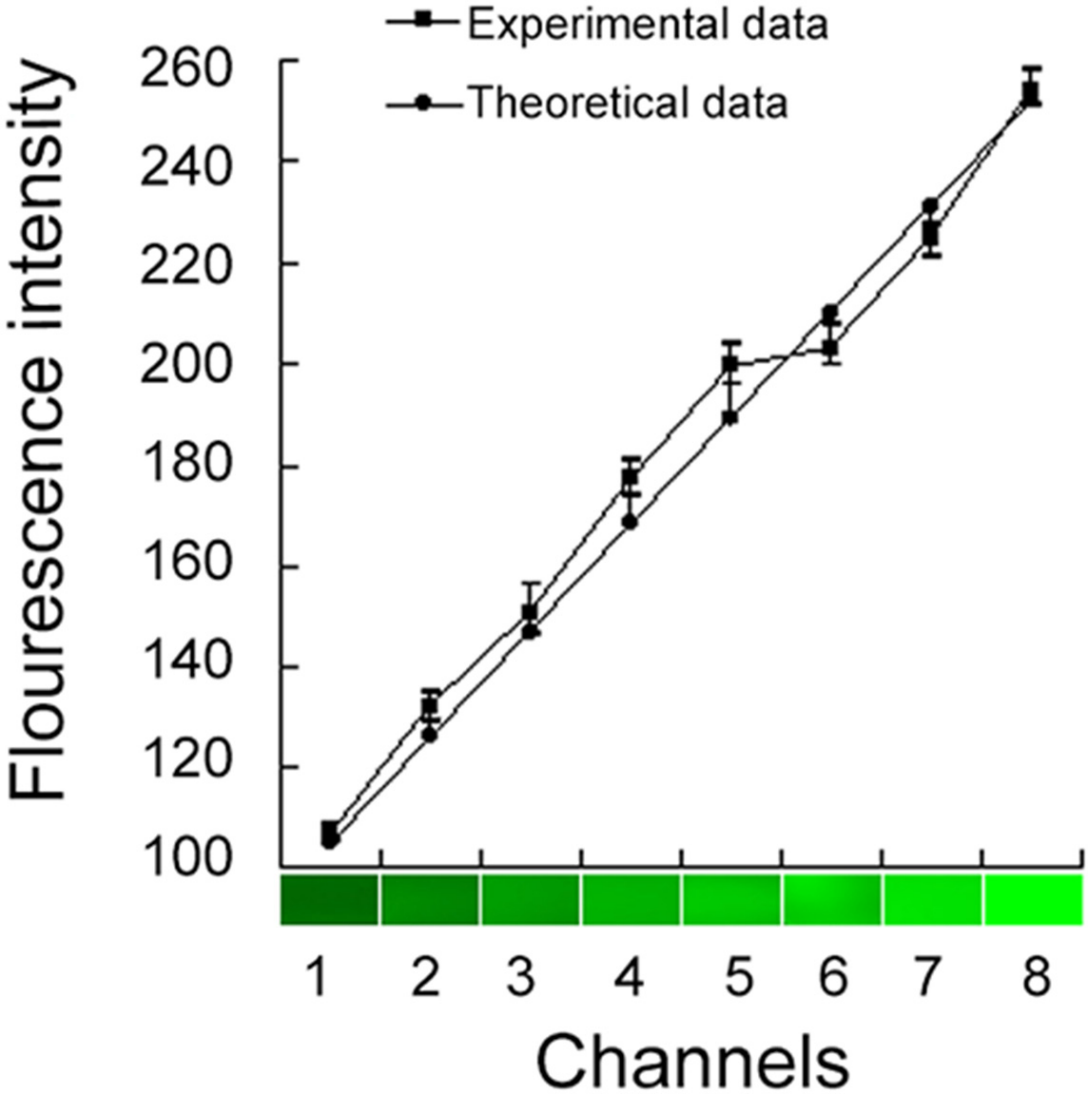
Comparison between experimentally measured fluorescence intensities at eight channels and theoretically predicted values.

To verify the concentration gradient generated by the concentration gradient generator, acridine orange and PBS were pumped into the microfluidic chip and the fluorescence intensities were measured at the eight outlets. The results (Fig 2) agreed satisfactorily (coefficient of correlation > 0.992) with the predicted linear pattern [23], indicating that the system could generate a reliable concentration gradient for cellular studies.

#### Effects of MTX and CMCS on survival of H460 cells cultured on the microfluidic chip

**Fig 3:**
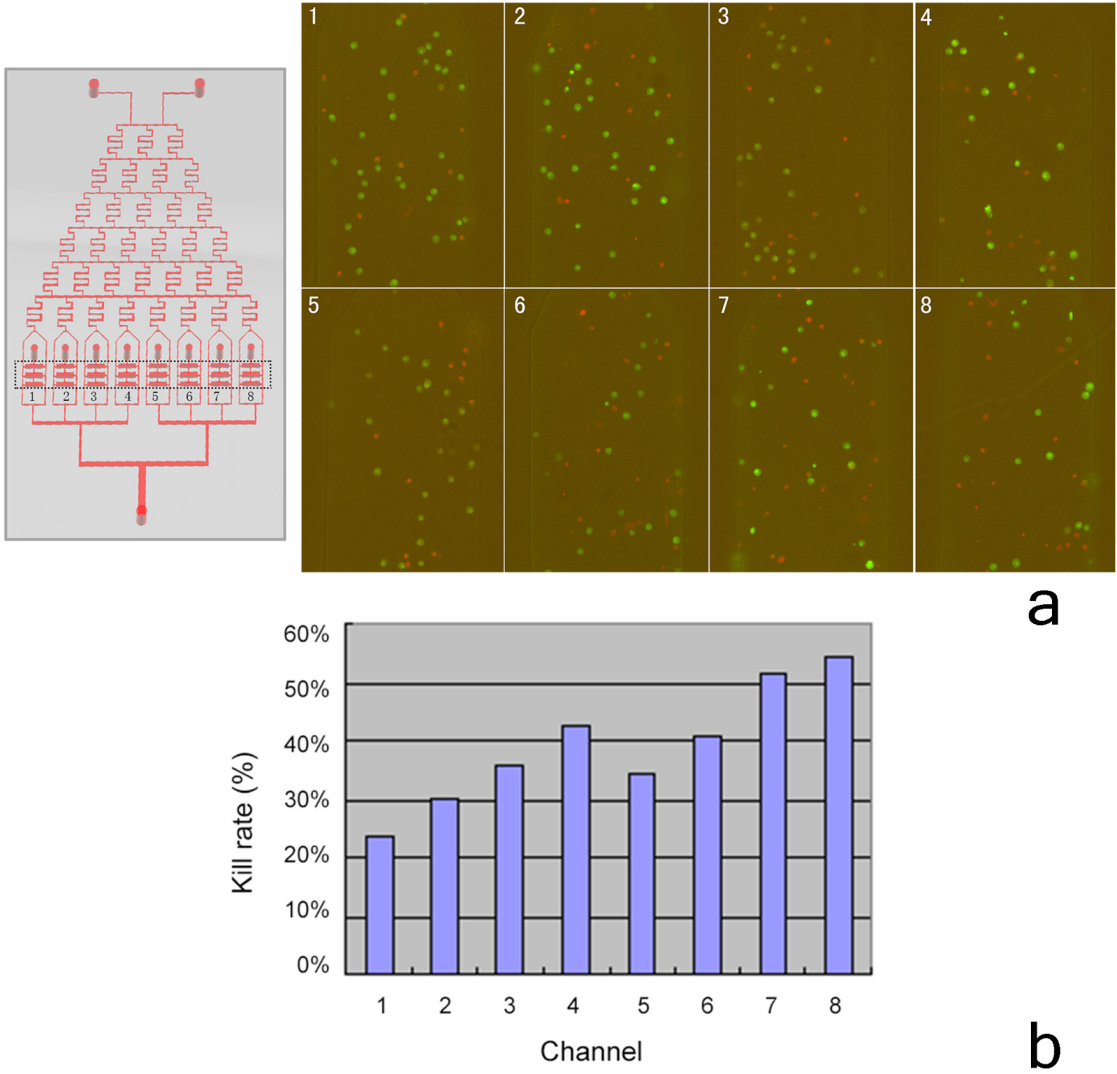
Killing of H460 cells cultured in eight channels in the microfluidic device; a) fluorescence micrographs; b) proportion of dead cells.

H460 cells were seeded in the cell culture unit of the system, and exposed to various MTX-CMCS concentration combinations (Table 1). After culture for 2 d, acridine orange/propidium iodide viability staining (Fig 3) showed that channel 4 (57 μg/mL CMCS + 21 μg/mL MTX) was associated with significantly lower H460 cell viability than channels 1, 2, 3, and 5, indicating it to be a suitable drug combination for further studies. The viability of channel 4 (57 μg/mL CMCS + 21 μg/mL MTX) and channel 6 (29 μg/mL CMCS + 36 μg/mL MTX) were similar (*P* = 0.175), but because channel 4 contained a lower MTX concentration, it was considered safer due to the adverse side effects of MTX. Although channels 7 (14 μg/mL CMCS + 43 μg/mL MTX) and 8 (50 μg/mL MTX) were associated with the lowest viability of H460 cells, they contained inappropriately high MTX concentrations (serving as positive controls) and were not suitable for further studies. Therefore, the concentrations of CMCS and MTX of channel 4 (57 μg/mL CMCS + 21 μg/mL MTX) were used in subsequent studies.

#### Effects of MTX and CMCS on H460 cells cultured in plates

**Fig 4:**
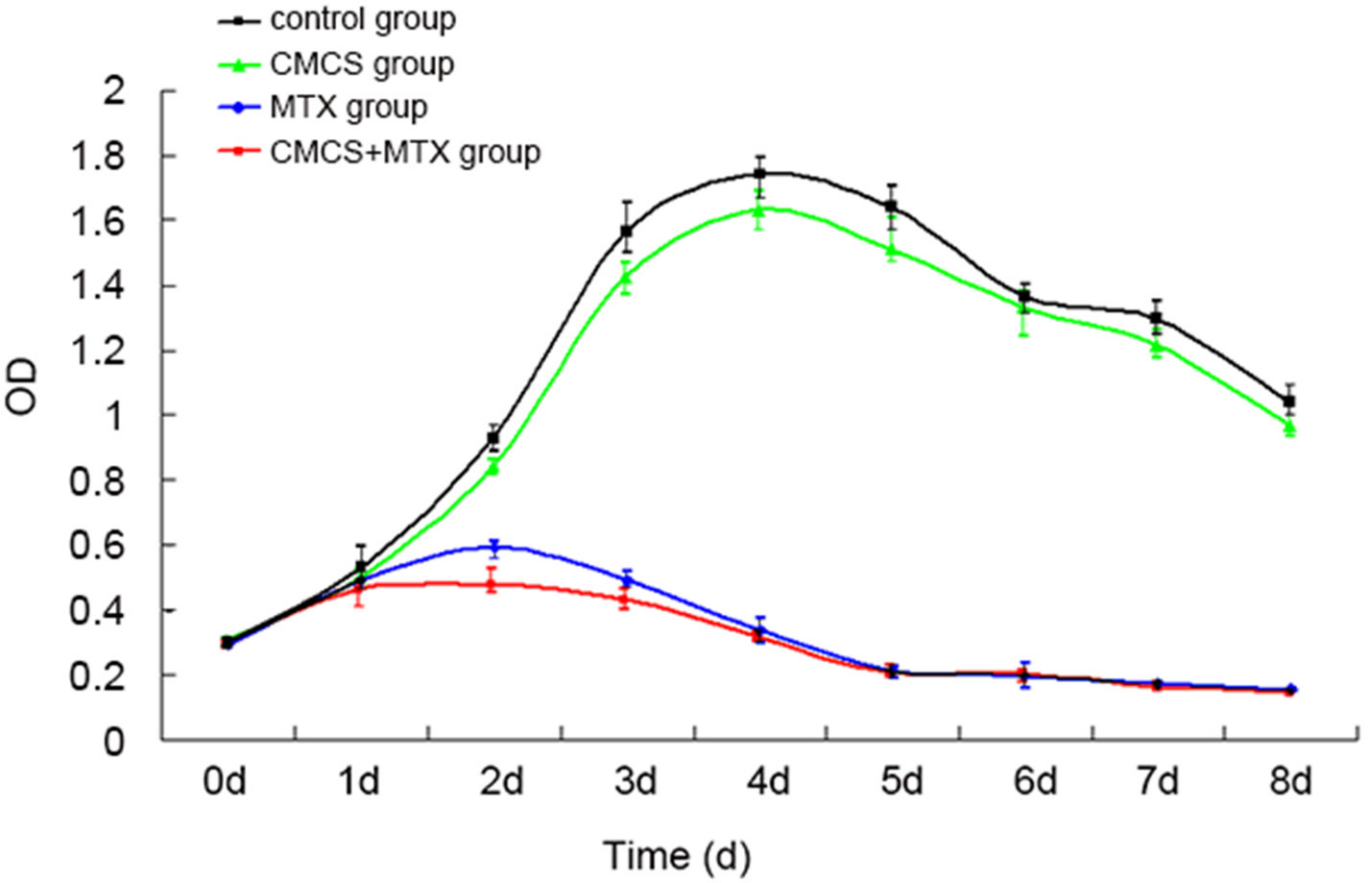
Growth curves of various groups under flask culture conditions.

To differentiate the effects of MTX and CMS on H460 cells, the cells were cultured in plates under four conditions: unsupplemented (blank control), 57 μg/mL CMCS, 21 μg/mL MTX, or 57 μg/mL CMCS + 21 μg/mL MTX, and growth curves were determined by MTT assays (Fig 4). The growth curve of the H460 cells in the CMCS group was generally similarly to that of the control group, but was consistently lower from day 1. On days 3, 4, 5, 7, and 8 (but not day 6; *P* = 0.234), the difference was statistically significant (all *P* < 0.05). This suggested that CMCS alone might have a moderate inhibitory effect on the survival or proliferation of these tumor cells. In addition, the growth curve of H460 cells in the MTX group was dramatically lower than that of the control group. Importantly, the growth curve of the H460 cells in the CMCS+MTX group was consistently lower than that of the MTX group between days 1 and 4, and the difference was statistically significantly on day 2 (*P* = 0.000), which may suggest a synergistic effect between the two compounds during the early stage of culture.

### Mechanical properties and microstructures of drug-loaded bone cement

#### Mechanical properties of drug-loaded cement

**Fig 5:**
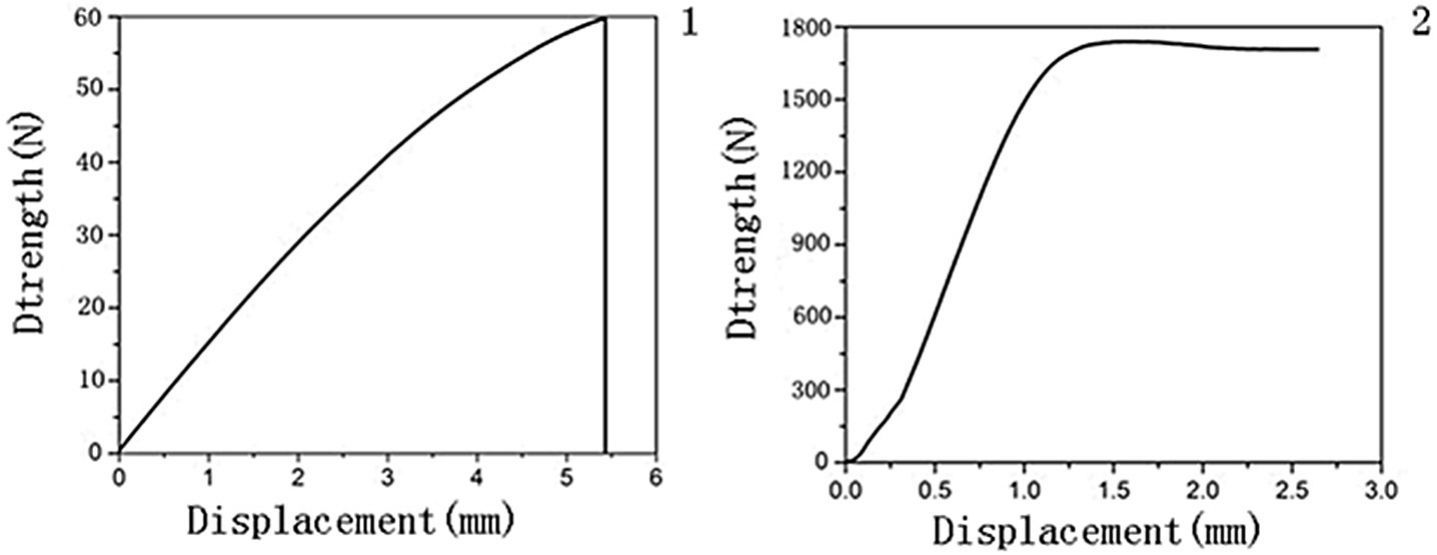
Representative stress strain curves.

**Fig 6:**
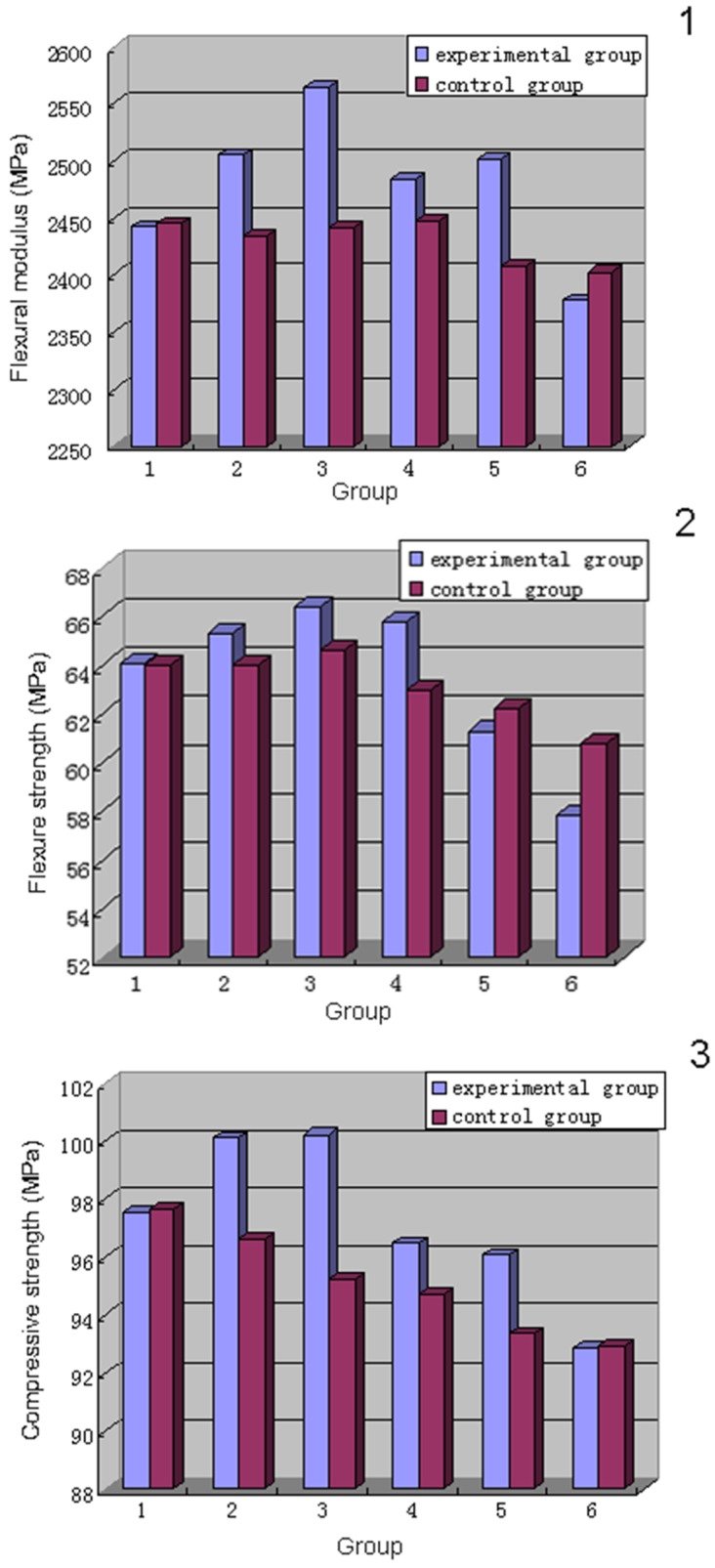
a) Bending moduli, b) bending strengths, and c) compressive strengths of various groups.

The effects of CMCS incorporation on the mechanical properties of cement were determined by four-point bending and compression tests [24] (Table 2; Fig 5). The bending modulus values (Fig 6A) of experimental groups 2, 3, and 5 (0.8% CMCS + 0.3% MTX, 1.6% CMCS + 0.6% MTX, and 3.2% CMCS + 1.2% MTX, respectively) were significantly higher than that of the corresponding control groups (containing MTX at the same concentrations). In contrast, the bending modulus values of experimental groups 1, 4, and 6 (0% CMCS + 0% MTX, 2.4% CMCS + 0.9% MTX, and 4% CMCS + 1.5% MTX) were similar to that of the corresponding controls (*P* = 0.944, 0.196, and 0.389).

Regarding inter-group comparisons, the bending modulus of experimental group 1 was significantly lower than that of group 3 (*P* = 0.012), similar to groups 2 and 4 (*P* = 0.174 and 0.057, respectively), and significantly higher than groups 5 and 6 (*P* = 0.003 and 0.000). Among the control groups, group 1 was significantly higher than group 6 (*P* = 0.001) and similar to groups 2–5 (*P* = 0.983, 0.468, 0.275, and 0.055).

The bending strength (Fig 6B) of experimental groups 3 (1.6% CMCS + 0.6% MTX) and 4 (2.4% CMCS + 0.9% MTX) were significantly higher than that of their corresponding control groups (*P* = 0.048, 0.003). Group 6 (4% CMCS + 1.5% MTX) was significantly lower than the control (*P* = 0.002). Groups 1, 2, and 5 (0% CMCS + 0% MTX, 0.8% CMCS + 0.3% MTX, and 3.2% CMCS + 1.2% MTX) were similar (*P* = 0.984, 0.143, and 0.294) to their controls.

Among the experimental groups, group 1 was significantly lower than group 3 in bending strength (*P* = 0.012), similar to groups 2 and 4 (*P* = 0.174 and 0.057, respectively), and significantly higher than groups 5 and 6 (*P* = 0.003 and 0.000). Among the control groups, group 1 was significantly higher than group 6 (*P* = 0.001) and similar to groups 2–5 (*P* = 0.983, 0.468, 0.275, 0.055).

The compressive strengths (Fig 6C) of experimental groups 2 (0.8% CMCS + 0.3% MTX), 3 (1.6% CMCS + 0.6% MTX), 4 (2.4% CMCS + 0.9% MTX), and 5 (3.2% CMCS + 1.2% MTX) were significantly higher than that of their corresponding control groups containing MTX at the same concentrations (*P* = 0.000, 0.000, 0.032, and 0.002, respectively), whereas experimental groups 1 and 6 (0% CMCS + 0% MTX and 4% CMCS + 1.5% MTX) were similar to the controls (*P* = 0.914 and 0.930).

Among the experimental groups, the compressive strength of group 1 (0% CMCS + 0% MTX) was significantly lower than that of groups 2 (0.8% CMCS + 0.3% MTX; *P* = 0.003) and 3 (1.6% CMCS + 0.6% MTX; *P* = 0.002), similar to groups 4 (2.4% CMCS + 0.9% MTX; *P* = 0.192) and 5 (3.2% CMCS + 1.2% MTX; *P* = 0.073), and significantly higher than group 6 (4% CMCS + 1.5% MTX; *P* = 0.000). Among the control groups, the compressive strength of group 1 (0% MTX) was similar to group 2 (0.3% MTX, *P* = 0.204) and significantly higher than groups 3–6 (0.6%-1.5% MTX; *P* = 0.005, 0.001, 0.000, and 0.000, respectively).

#### Microstructure of drug-loaded cement

**Fig 7:**
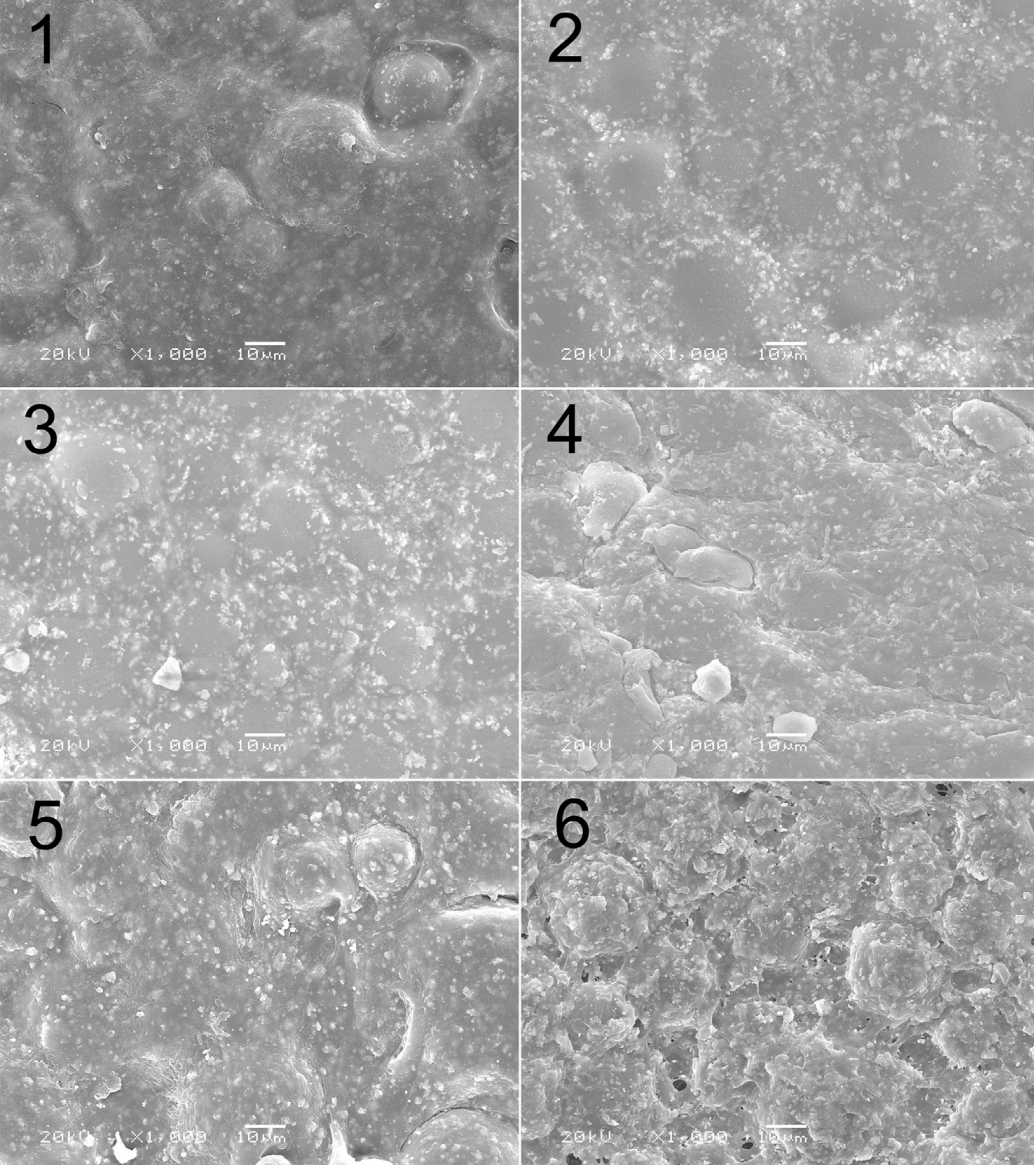
Scanning electron micrographs showing microstructures of various samples.

SEM revealed that the cement sample of experimental group 1 (0% CMCS + 0% MTX; Table 2) had a homogeneous microstructure and density, with a smooth surface, shallow circular grooves, and regularly arranged crystals (Fig 7). The surfaces of the cement samples of experimental groups 2 (0.8% CMCS + 0.3% MTX) and 3 (1.6% CMCS + 0.6% MTX) had particles, which were likely drug powders. The circular grooves were more superficial and almost indiscernible, compared with experimental group 1. Overall, the microstructural homogeneity of experimental groups 2 and 3 appeared better than that of group 1, potentially explaining the significantly higher compressive strength of these groups compared with group 1 (Fig 6). Experimental groups 4 (2.4% CMCS + 0.9% MTX) and 5 (3.2% CMCS + 1.2% MTX) showed more white particles and deeper circular grooves compared with groups 2 and 3, good structural homogeneity, and similar crystal alignment to experimental group 1. Moreover, the grooves became interconnected, creating isolated islands of raised cement across the surface.

#### Effect of CMCS incorporation on MTX release from bone cement

**Fig 8:**
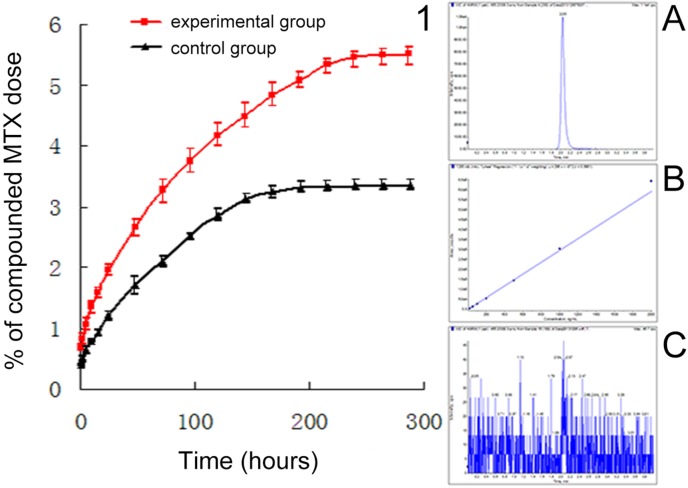
Effect of CMCS incorporation on the rate of MTX release from bone cement.

The incorporation of CMCS (Fig 8) delayed the time for MTX release to reach the plateau, and increased the proportion released at the plateau. The control group (cement containing 1.5% MTX alone) reached a plateau on day 7, having released 3.24% of the loaded MTX. In comparison, the experimental group (cement containing 4% CMCS + 1.5% MTX) plateaued on day 9, having released 5.34% of the MTX load. The rank sum test found that, at each timepoint, the released dose recorded from the experimental group was greater than that of the corresponding control group (all *P* = 0.029).

### *In vivo* evaluation of MTX-PMMA cement implants containing CMCS

#### *In vivo* implantation

**Fig 9:**
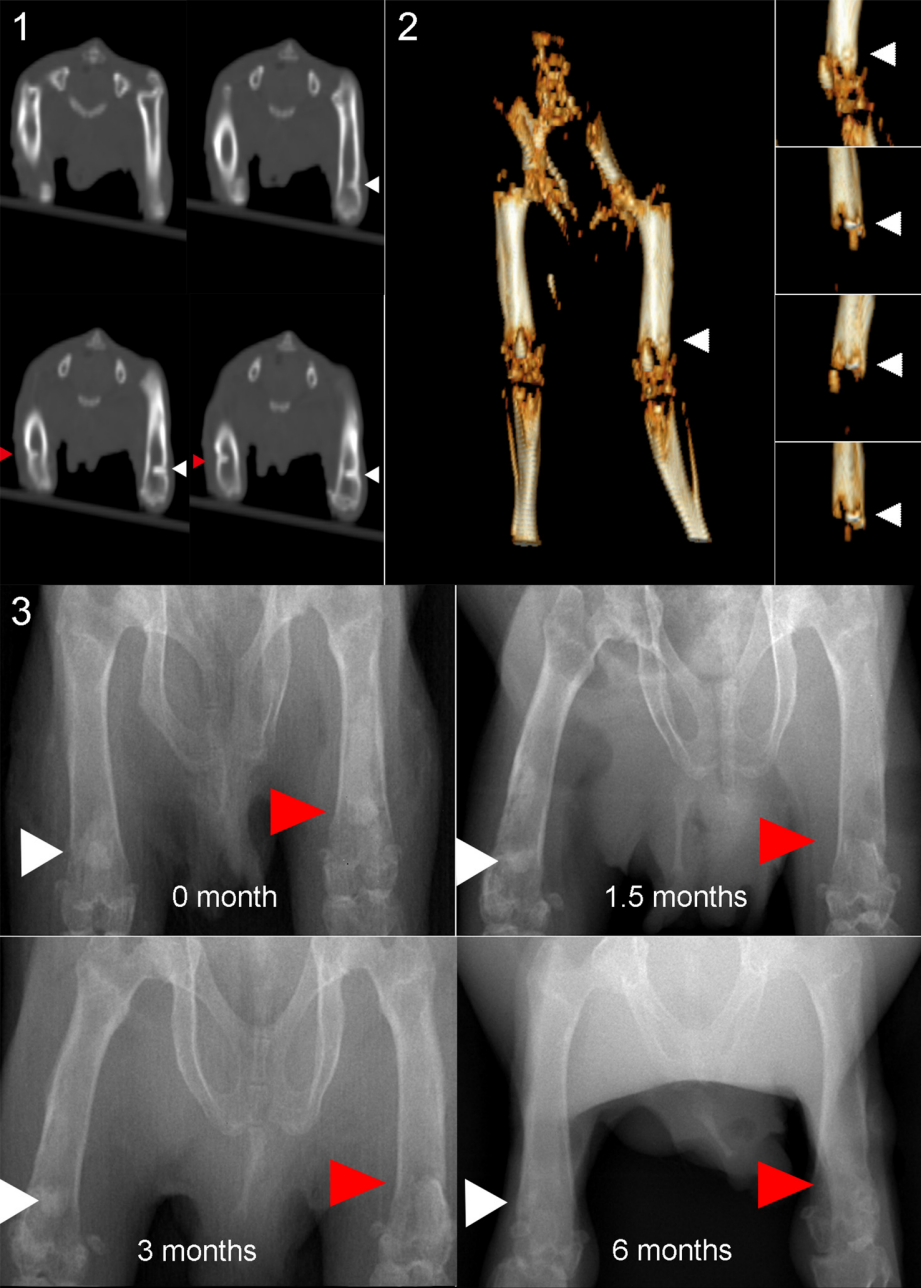
Computed tomography (CT) and radiography images showing cement sample integration with the host bone; a) CT image taken immediately after operation; b) 3D reconstructed CT image immediately after operation; c–f) radiographs taken 0, 1.5, 3, and 6 months, respectively.

The microstructural changes and interfacial integration of MTX-PMMA cement implants containing CMCS were evaluated relative to implants containing only MTX (control) in the right and left femurs of adult guinea pigs, respectively. Spiral CT and radiography (Fig 9) performed immediately after implantation revealed that the two types of bone cement implants were positioned symmetrically at similar depths, and a clear interface with the host bone was observed.

At 1.5 to 3 months after implantation, radiography revealed that the geometry of the control implants remained generally unchanged, and retained a clear interface with the adjacent bone. In comparison, the appearance of the experimental implants became gradually indistinct with a loss in density, and the interface with the adjacent bone became unclear. Six months after implantation, the experimental implants were less clear on radiographs, compared with the control implants. This indicated that, as the CMCS degraded in the experimental implants, the implants gradually decreased in density and integrated with the adjacent bone. However, more studies are needed to better characterize the *in vivo* fate of the experiment implants.

### Evaluation using SEM

**Fig 10:**
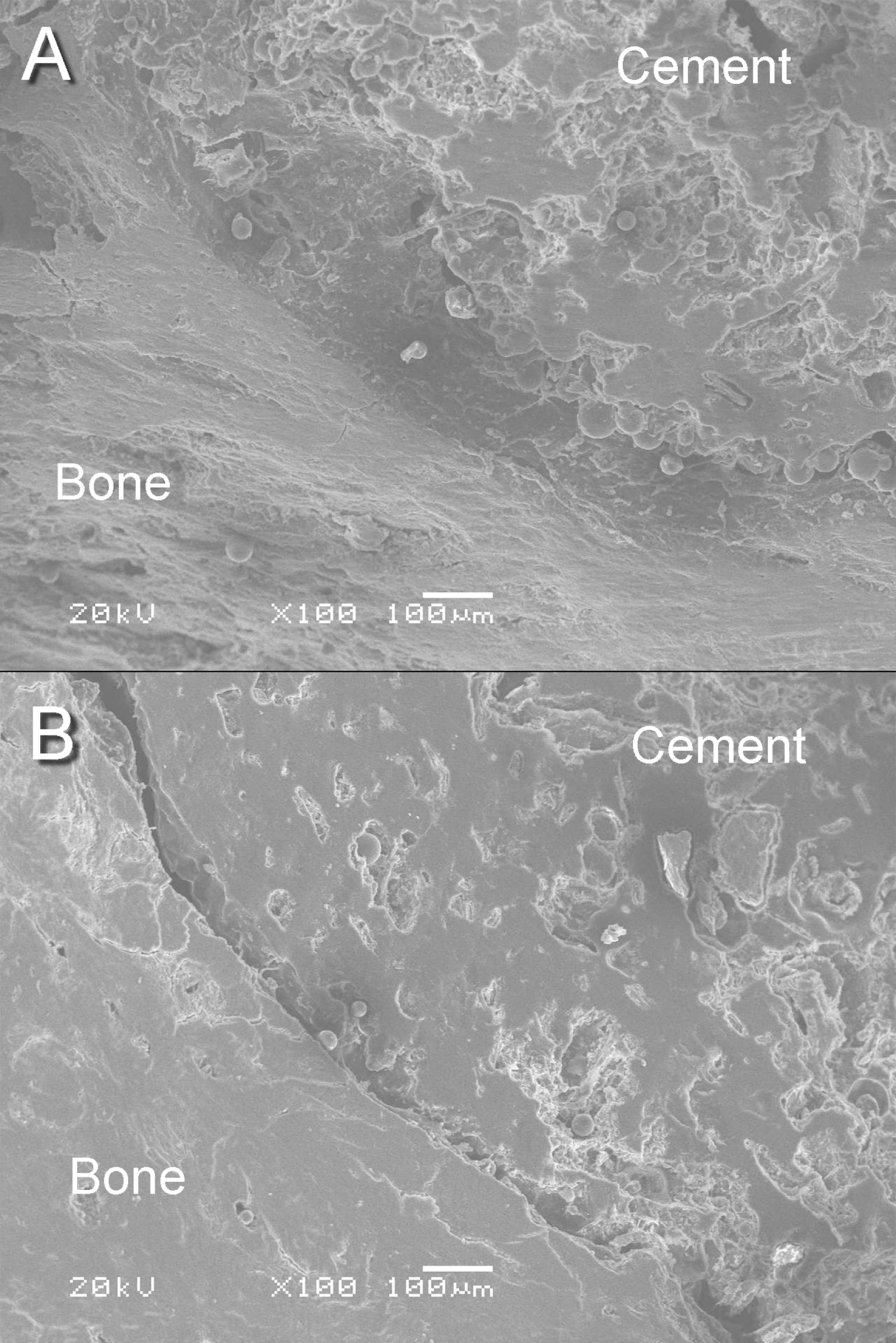
Scanning electron micrographs of a) control implant and b) experimental implant retrieved six months after implantation.

Six months after the guinea pigs were given the MTX-PMMA cement implants, with and without CMCS, the femurs were inspected using SEM. The control implant (without CMCS; Fig 10A) had a smooth and compact surface, indicative of a dense structure. The implant was clearly separated from the adjacent bone tissue by a gap, evidence of a lack of bone-implant integration. In comparison, the experimental implant with CMCS (Fig 10B) had a more porous surface. The adjacent bone was homogeneous in microstructure, and the implant had integrated closely with the adjacent bone tissue.

## Discussion

The objectives of this study were to examine the effect of CMCS on the effect of MTX in killing H460 cells and the influence of CMCS incorporation on the mechanical properties, *in vitro* MTX release, and *in vivo* response of PMMA cement. The on-chip culture showed that 57 μg/mL combined with 21 μg/mL MTX resulted in a higher killing rate than other combinations.

Flask culture found that, compared with cells exposed to 21 μg/mL MTX alone, cells simultaneously exposed to 57 μg/mL CMCS and 21 μg/mL MTX had significantly lower viability. Incorporation of CMCS to PMMA cement significantly improved the bending modulus and the compressive strength. Incorporation of 4% (w/w) CMCS also delayed the time-to-plateau of MTX release and increased the fraction released at the plateau. *In vivo* implantation indicated that PMMA cement containing 4% CMCS and 1.5% MTX integrated with the host bone of guinea pig femur better than those containing 1.5% MTX alone. Collectively, these results show that incorporation of an appropriate amount of CMCS to MTX-PMMA bone cement can produce three advantages, as discussed below.

### CMCS enhancement of tumor cell destruction by MTX

In our study, an effective MTX-CMCS concentration combination was screened by cell culture on a microfluidic chip. This on-chip experiment, however, only represents the effect of this concentration combination at a single timepoint. Therefore, a flask culture was performed to understand the effect of that combination on the growth of H460 cells. From day 2 (Fig 4), the four groups started to show statistically significant differences. The CMCS+MTX group was the most effective in reducing H460 cell viability, followed by the MTX group, and then the CMCS group. The peak killing effect in the CMCS+MTX and MTX groups was observed on day 4. After day 5, the two groups behaved almost identically, indicating complete inhibition of cell survival in both groups. In addition, the CMCS and control groups were similar in profile, with the cell viability recorded from the former consistently lower than the latter. This moderate but consistent difference suggests that CMCS may have a moderate inhibitory effect on H460 cells.

We used an established method [25, 26] to verify whether a synergistic effect occurred between MTX and CMCS. In the method, a parameter *Q* representing the relationship between the two drugs is calculated using:

$$Q=E_{a}{{}_{+}}_{b}/(E_{a}+E_{b}-E_{a}\times E_{b})$$

Where *E*_*a+b*_ represents the killing effect obtained by simultaneously exposing cells to two drugs (denoted as *a* and *b*), and *E*_*a*_ and *E*_*b*_ represent effects obtained by exposing cells to each drug alone. In this method, *Q* > 2.0 indicates a significant synergistic enhancement, 1.15 < *Q* < 2.0 an observable synergistic enhancement, 0.85 < *Q* < 0.55 an observable antagonism, and *Q* < 0.55 is a significant antagonism.

Calculation found a *Q*-value of 1.16 for day 2, indicating an observable synergistic enhancement between MTX and CMCS at this stage. Actually, simultaneous exposure to MTX and CMCS produced a 12.09% lower viability than exposing cells to MTX alone (Fig 4). This synergistic effect suggests that by introducing an appropriate dose of CMCS, an equivalent number of cancer cells may be destroyed with a reduced MTX dose. This may reduce the local overshoot of MTX concentration, thereby minimizing the associated side effects of MTX such as impaired wound healing and toxic effects. The synergistic effect may be particularly pronounced at the early stage of treatment. In addition, the results suggest that CMCS intervention alone may affect tumor cell growth.

### Improvement of physicochemical properties of bone cement by CMCS

The body’s skeletal system constantly experiences complex mechanical stresses such as bending and compression. Consequently, mechanical properties are important factors in evaluating the function of bone cement. Our mechanical tests (Fig 5) found that, when the overall drug concentration (CMCS wt%+MTX wt%) in the bone cement was below 3%, the mechanical properties increased with increasing MTX concentration. For example, compared with control group 1, the bending modulus of experimental group 3 was increased by 5%, bending strength by 2.8%, and compressive strength by 5.2%. When the overall drug concentration was 3–5% (control/experimental groups 4 and 5), the mechanical properties changed minimally or increased moderately (compared with the control group 1) with the introduction of CMCS. When the concentration was 5.5% (experimental groups 6), the mechanical properties decreased (compared with the control group 1) with CMCS introduction. Interestingly, when <2% was incorporated in bone cement, the mechanical properties were even better than the drug-free (blank) cement (experimental group 1 cf. control group 1; Table 2 and Fig 5).

These changes in mechanical properties may be related to microstructure. SEM showed that, when the overall drug concentration was 0–3%, the cement surface was homogeneous and dense. Fine particles and shallow grooves appeared. With the combined drug concentration at 5.5% (experimental group 6), the grooves were deeper and more numerous. These microstructural changes are consistent with the results of the mechanical tests.

CMCS is a polar macromolecule of high viscosity. Therefore, when incorporated at an appropriately low concentration, CMCS serves as a binder to join cement particles during mixing. However, when introduced at a high concentration, CMCS (a macromolecule occupying a large space) may adversely affect the structure of the cement and, thus, its mechanical properties. Hence, improved mechanical properties were obtained only at reasonably low CMCS concentrations.

Earlier studies [27] reported the longitudinal and transverse compressive strengths of human long bones to be 131–224 MPa and 106–133 MPa respectively, and the compressive strength and rotary tensile strength of human cancellous bones were 30 MPa and 2.1 MPa. Moreover, the mechanical properties of medical-grade PMMA bone cement are: compressive strength ≥70 MPa, bending modulus ≥1800 MPa, and bending strength ≥50 MPa (ISO standard 5880: 2002). The values recorded in the current study easily exceed the standard requirements. Therefore, incorporation of CMCS and MTX would not sacrifice the mechanical properties of the bone cement.

Results of the *in vitro* release analysis showed that CMCS extended the time-to-plateau from 7 to 9 days, and increased the proportion of MTX released at plateau from 3.24% to 5.34%. CMCS features a large molecular mass and carries hydrophilic carboxymethyl groups. After dissolution, CMCS forms a polyelectrolyte solution. MTX is insoluble in water and thus interacts intimately with hydrophobic groups in CMCS. In an aqueous environment, the hydrophilic groups of CMCS face outward while the hydrophobic groups face inward to encapsulate MTX. By forming such capsules, CMCS facilitates the release of MTX from bone cement. In addition, the dissolution of CMCS and MTX from the superficial surface of the cement creates channels in the cement matrix, providing pathways for the further release of MTX in the deeper layer [28]. This may overcome the limited MTX release arising from the dense structure of PMMA cement.

The incorporation of CMCS is expected to allow a prolonged stable MTX release, thereby enabling sustained tumor destruction. On the other hand, because PMMA cement typically releases only a small fraction of the loaded anti-tumor drug, clinicians often have to increase the amount of drug loaded in the cement. An inappropriately high drug load, however, may compromise the mechanical properties and increase the risk of failure *in vivo*. The current study found that the introduction of CMCS increased the proportion of the drug that can be released from the cement. This could increase the drug available for therapy without compromising the mechanical properties of the cement.

From another perspective, CMCS higher than a threshold concentration can reduce the mechanical properties of the bone cement. In some cases, this may be desirable. For some osteoporotic patients, an appropriate reduction of bending modulus may prevent overloading of the adjacent bone and prevent fractures. Moreover, elevated CMCS concentration also increases the porosity and the proportion of drug available for released. Therefore, high strength is not the only criterion in the development of drug-containing cement [29]. Rather, both the patient and disease conditions should be considered when designing the cement.

### Improved cement-bone integration

After implantation in guinea pig femurs, compared with implants containing MTX alone, in those containing MTX and CMCS the delineation of the bone-implant interface was less obvious, suggesting a better integration with the adjacent bone. SEM clearly revealed that, 6 months after implantation, the implants containing MTX and CMCS had become porous [2]. It is generally accepted that higher porosity results in better integration with bone [19]. For example, studies on synthetic HA implants have found that when the micropore diameters are >100 μm, capillary vessels can grow into the pores allowing inward migration of bone cells, thereby leading to bone ingrowth and a better supply of nutrients [28]. Tissue growth into pores stabilizes the cement and reduces disadvantages associated with conventional cements that lack pores, disadvantages that include poor tissue integration and eventual implant loosening [30]. In addition to effective mechanical augmentation, guided tissue ingrowth and satisfactory cement-bone integration [31,32] are the goals of an ideal bone cement.

## Conclusion

The incorporation of CMCS into MTX-PMMA cement synergistically increased the ability of MTX to kill H460 cells. CMCS incorporation also improved the mechanical properties of the cement, and prolonged the time to plateau of MTX release. After implantation in guinea pig femurs, cement implants containing CMCS formed surface pores and thereby improved implant-bone integration and implant fixation. Therefore, CMCS incorporation is a simple and feasible method to benefit the performance of PMMA cement for new clinical applications.

## Supporting Information

S1 File Additional details of post-operative care, efforts to alleviate suffering (including anaesthesia and/or analgesia), and the method of euthanasia. (DOC)
