# Supplementary material for: Effects of Incorporating Carboxymethyl Chitosan into PMMA Bone Cement Containing Methotrexate
Source: PLoS One. 2015 Dec 14;10(12):e0144407. doi: 10.1371/journal.pone.0144407 (PMC4690603; doi:10.1371/journal.pone.0144407)
Supplement: S1 File — (DOC) [file pone.0144407.s001.doc]

**Additional details on animal care and handling**

**Postoperative care**

After operation, each animal was placed in a separate cage with free access to water and food. The cage was cleaned and water refreshed daily. The animal was given diet manufactured for guinea pigs supplemented with fresh vegetable leaves.

The animal received intraperiotoneal injection of penicilline for 3 d (80 K units/injection, one injection/day; injection fluid prepared by dissolving 80K units in 0.1 ml of a 0.9% NaCl solution). The wound was disinfected daily with iodophore and erythromycin ointment was applied over the wound to prevent infection.

The food/water intake and defecation of each animal was regularly observed to evaluate its recovery. Two weeks after operation, the wounds in all animals healed and the suture was removed.

**Euthanasia**

Six weeks after operation, the animal was euthanized by intraperitoneal injection of an overdose of 20% chloral hydrate (1000 mg/kg body weight). After experiment, the animal bodies were placed in biowaste bags. The bags were labeled, sealed, and incinerated in a specialized biowaste/biohazard incinerator following standard procedures.
